# Arsenic trioxide and ascorbic acid interfere with the BCL2 family genes in patients with myelodysplastic syndromes: an ex-vivo study

**DOI:** 10.1186/1756-8722-5-53

**Published:** 2012-09-10

**Authors:** Sara Galimberti, Francesca Guerrini, Flavia Salvi, Iacopo Petrini, Daniela Gioia, Emanuela Messa, Giuseppe A Palumbo, Daniela Cilloni, Mario Petrini, Alessandro Levis

**Affiliations:** 1 Department of Oncology, Transplant, New Advances in Medicine, Section of Hematology, University of Pisa, Pisa, Italy; 2 S.O.C. di Ematologia, Azienda Ospedaliera SS Antonio e Biagio, Alessandria, Italy; 3 Department of Oncology, Transplant and New Advances in Medicine, BIOS, University of Pisa, Pisa, Italy; 4 Department of Clinical and Molecular Bio-Medicine, Section of Hematology, Oncology and General Pathology, Catania, Italy; 5 Department of Clinical and Biological Sciences, University of Turin, Turin, Italy; 6 Division of Haematology, Department of Oncology, Transplant and Advances in Medicine, University of Pisa, Via Roma 67, Pisa, 56126, Italy

**Keywords:** ATO, Ascorbic acid, Myelodysplastic syndromes, MDS, Apoptosis

## Abstract

**Background:**

Arsenic Trioxide (ATO) is effective in about 20% of patients with myelodysplasia (MDS); its mechanisms of action have already been evaluated in vitro, but the in vivo activity is still not fully understood. Since ATO induces apoptosis in in vitro models, we compared the expression of 93 apoptotic genes in patients’ bone marrow before and after ATO treatment. For this analysis, we selected 12 patients affected by MDS who received ATO in combination with Ascorbic Acid in the context of the Italian clinical trial NCT00803530, EudracT Number 2005-001321-28.

**Methods:**

Real-time PCR quantitative assays for genes involved in apoptosis were performed using TaqMan® Assays in 384-Well Microfluidic Cards “TaqMan® Human Apoptosis Array”.

Quantitative RT-PCR for expression of EVI1 and WT1 genes was also performed. Gene expression values (Ct) were normalized to the median expression of 3 housekeeping genes present in the card (18S, ACTB and GAPDH).

**Results:**

ATO treatment induced up-regulation of some pro-apoptotic genes, such as HRK, BAK1, CASPASE-5, BAD, TNFRSF1A, and BCL2L14 and down-regulation of ICEBERG. In the majority of cases with stable disease, apoptotic gene expression profile did not change, whereas in cases with advanced MDS more frequently pro-apoptotic genes were up-regulated. Two patients achieved a major response: in the patient with refractory anemia the treatment down-regulated 69% of the pro-apoptotic genes, whereas 91% of the pro-apoptotic genes were up-regulated in the patient affected by refractory anemia with excess of blasts-1. Responsive patients showed a higher induction of BAD than those with stable disease. Finally, WT1 gene expression was down-regulated by the treatment in responsive cases.

**Conclusions:**

These results represent the basis for a possible association of ATO with other biological compounds able to modify the apoptotic pathways, such as inhibitors of the BCL2 family.

## Introduction

Arsenic Trioxide (ATO) is an ancient drug that, in the most recent years, has been rediscovered and evaluated for the treatment of promyelocytic leukemia, multiple myeloma, and myelodysplastic syndromes (MDS) [1].

In MDS, two phase-II multicenter trials have reported interesting results, either in low- or high-risk patients [2,3], with hematological improvement rates of 20-30%.

More recently, arsenic trioxide has been used in combination with thalidomide and retinoic acid in high-risk MDS patients, resulting in response rate of 48% and efficacy of 25% [4].

ATO exerts its anticancer activity inducing apoptosis through the disequilibrium of apoptotic/anti-apoptotic BCL2 family members [5,6] cytochrome C release, loss of mitochondrial transmembrane potential, reactive oxygen species generation [1], inactivation of NF-kB [7,8], and activation of caspases [9]. Moreover, an anti-angiogenetic activity of ATO has also been reported [10]. Because stable MDS cell lines are still lacking, PML-RARa-negative acute myeloid leukemia HL60 cells are frequently adopted as in vitro models. Recently, our group reported synergistic effects for the combination of the proteasome inhibitor Bortezomib and ATO in HL60 cell line [11].

However, the in vivo mechanisms of action of ATO in MDS patients are still matter of debate. Since ATO induces apoptosis in vitro, we evaluated the expression of 93 apoptotic genes in patients’ bone marrow before and after ATO plus Ascorbic Acid treatment, in the context of the Italian clinical trial NCT00803530, EudracT Number 2005-001321-28.

In addition, the expression of WT1 and EVI1 has been evaluated and correlated with those of the apoptotic genes, because of WT1 and EVI1 prognostic value in MDS [12,13].

## Patients and methods

### Patients

Twelve MDS patients receiving ATO plus Ascorbic Acid in the context of the clinical trial NCT00803530, EudracT Number 2005-001321-28 were selected for this molecular characterization. For these patients, RNA collected from the bone marrow immediately before the first administration of the treatment was available.

ATO was administered at a dosage of 0.3 mg/Kg during the first week of therapy, and at a dosage of 0.25 mg/Kg during the subsequent weeks (week 2 to 16); Ascorbic Acid was administered at 1000 mg IV within 30 minutes after each arsenic trioxide infusion, for 16 consecutive weeks.

Table 1 summarizes patients’ characteristics. Three mL of bone marrow blood were collected in an EDTA tube by needle aspiration just before the beginning of the treatment and after the last administration.

**Table 1:** Patients' characteristics

**Characteristics**	**Patients**	
	**No.**	**%**
Total patients enrolled	12	
Median age, years	69	
Sex		
Male	7	58
Female	5	42
IPSS risk score		
Low	0	0
Intermediate 1	7	59
Intermediate 2	1	8
High	4	33
WPSS risk score		
Very low	1	8
Low	3	25
Intermediate	0	0
High	5	42
Very high	3	25
WHO classification		
RA	3	25
RARS	0	0
RCMD	0	0
RCMD-RS	0	0
RAEB1	5	42
RAEB2	4	33
Transfusion dependence at baseline		
RBC only	8	67
PLT only	1	8
RBC + PLT	1	8
No	2	17
Cytopenias at baseline		
0	0	0
1	7	58
2	5	42
3	0	0
Hematological median values at enrollment		
N (x10^9^/L)	2,04	
Hb (g/dL)	8.9	
PLT (x10^9^/L)	174	
Karyotype		
Normal	5	42
Complex	2	17
+8	1	8
Del7	1	8
Other abnormalities	3	25

RA = refractory anemia, RARS = refractory anemia with ringed sideroblasts, RCMD = refractory cytopenia with multilineage dysplasia, RCMD-RS = refractory cytopenia with multilineage dysplasia and ringed sideroblasts, RAEB-1 = refractory anemia with excess of blasts-1, RAEB-2 = refractory anemia with excess of blasts-2.

Patients were diagnosed in agreement with WHO criteria and classified according to the International Prognostic Scoring System (IPSS) and the WHO classification-based prognostic scoring system (WPSS), as previously described [14,15]. Treatment responses were assessed according to the International Working Group (IWG) criteria [16].

This study has been conducted in accordance with the Declaration of Helsinki, and all patients gave informed consent for the clinical trial and this molecular characterization.

### Gene expression assays

RNA was extracted from bone marrow blood using RNeasy Mini Kit (QIAGEN, Valencia, CA, USA). Real-time PCR quantitative assays for genes involved in apoptosis were performed using TaqMan® Assays in 384-Well Microfluidic Cards “TaqMan® Human Apoptosis Array” (Catalog Number 4378701, Applied Biosystems, CA, USA). Real-time PCR was performed in a 7900HT Real-Time PCR System (Applied Biosystems) and data were analyzed using SDS 2.2 software.

Quantitative RT-PCR expression of EVI1 gene was performed as previously described [13]; quantitative WT1 expression was assessed by using the “WT1 ProfileQuant® kit” (Ipsogen, Marseille, France).

### Statistical analysis

Gene expression was analyzed using GenespringGx 11.5 (Agilent, Palo Alto, CA, USA). Gene expression values (Ct) were normalized to the median expression of three housekeeping genes present in the card (18S, ACTB and GAPDH). Normalized gene expression (ΔCt) before and after treatment was compared using two tails paired t-test. Differential gene expression before and after treatment was calculated (−ΔΔCt) and differences between patients who experienced a response or not (stable disease vs major or minor responses) were evaluated by an unpaired t-test.

Ingenuity pathway analysis was adopted to explore the interaction between differently expressed genes.

To determine significances in categorized variables, SPSS 17.0 (SPSS Inc, Chicago, IL, USA) was used to calculate Pearson Chi-square or Fisher’s exact test, when appropriate. All tests were 2-sided. Results were considered statistically significant for p ≤ 0.05.

## Results

### Patients characteristics

Characteristics of patients enrolled in this study are listed in the Table 1. Note that the purpose of this article was not the presentation of results arising from the clinical trial; of consequences, the characteristics listed in the Table are not fully representative of the whole series of patients.

Three patients were affected by refractory anemia (RA), 5 by refractory anemia with excess of blasts-1 (RAEB1), and other 4 by refractory anemia with excess of blasts-2 (RAEB2). IPSS was intermediate-1 in 7 cases, intermediate-2 in one patient, and high in the remaining 4. Eight patients required red blood cell transfusion, one only platelet transfusion, and one both red blood cells and platelets support. The WPSS score was very low in one case, low in 3, high in 5, and very high in other 3 patients.

### Apoptotic genes expression

First, we analyzed the gene expression profile of patient #12, considered as the “test patient”; indeed, expression levels of apoptotic and anti-apoptotic genes were analyzed the day before receiving arsenic trioxide and the day after the end of the loading phase (at day 6). This patient, affected by refractory anemia with excess of blasts-1, with IPSS intermediate-1 risk score, achieved a major response.

After treatment, 18 genes resulted up-regulated in this patient (14/18 = 78%, pro-apoptotic), and only 3 genes down-regulated (2 anti-apoptotic; Table 2).

**Table 2:** Results from Quantitative RT-PCR assays in pt #12 (the test patient)

**Gene identification**		**Gene function**	**2-ΔΔCt**
**BAK1**	**Hs00832876_g1**	**Pro-apoptosis**	**36.1**
BAX	Hs00751844_s1	Pro-apoptosis	5.5
BCL2	Hs00608023_m1	Anti-apoptosis	3.2
BIM	Hs00708019_s1	Pro-apoptosis	4.4
**BCL2L14**	**Hs00373302_m1**	**Pro-apoptosis**	**21.6**
BIRC1	Hs01847653_s1	Anti-apoptosis	10.7
BIRC4	Hs00745222_s1	Anti-apoptosis	8.1
**BIRC8**	**Hs01057786_s1**	**Anti-apoptosis**	**521.7**
BNIP3	Hs00969291_m1	Pro-apoptosis	15.7
BOK	Hs00261296_m1	Pro-apoptosis	5.4
**CASPASE-5**	**Hs00362072_m1**	**Pro-apoptosis**	**18.2**
DEDD2	Hs00370206_m1	Pro-apoptosis	2.8
**HRK**	**Hs00705213_s1**	**Pro-apoptosis**	**338.8**
LTA	Hs99999086_m1	Pro-apoptosis	13.1
LTB	Hs00242739_m1	Pro-apoptosis	7.1
TNFRSF1A	Hs01042313_m1	Pro-apoptosis	3.6
TRAIL	Hs00234356_m1	Pro-apoptosis	3.8
TRADD	Hs00601065_g1	Pro-apoptosis	3.4
BCL2L10	Hs00368095_m1	Pro-apoptosis	0.32
BIRC5	Hs00977611_g1	Anti-apoptosis	0.41
BIRC7-LIVIN	Hs00223384_m1	Anti-apoptosis	0.38

2-ΔΔCt lists how many times a gene is up- or de-regulated in bone marrow after ATO + ascorbic acid after treatment versus before therapy.

Among the pro-apoptotic genes, those with higher increasing rate were HRK, BAK1, BCL2L14, and CASPASE-5.

**Figure 1:**
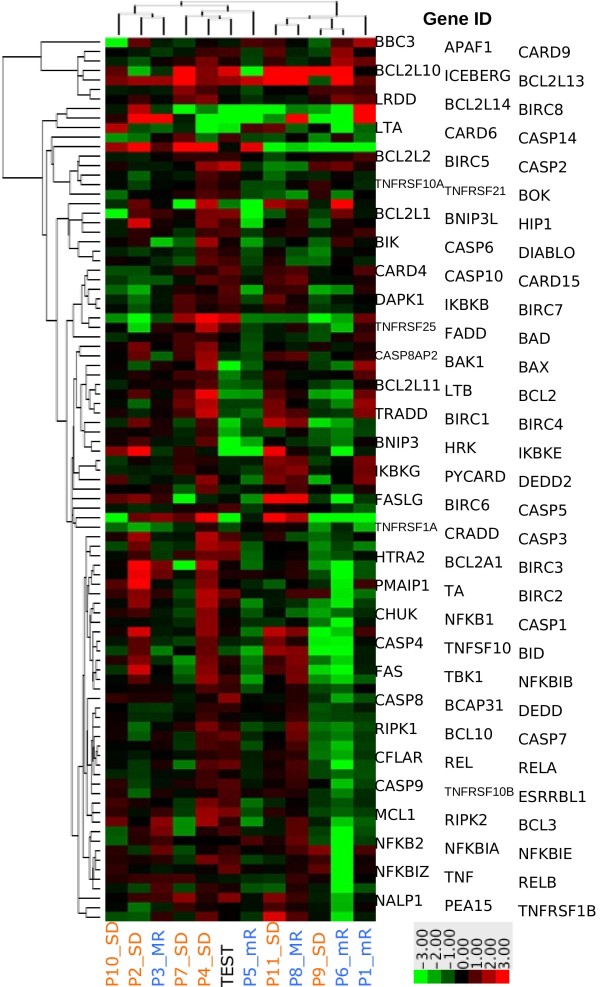
**Unsupervised cluster analysis of apoptotic gene expression modification upon ATO and Ascorbic Acid treatment.** Figure depicts -ΔΔCt of the “test patient” (lane #6) versus the remaining ones.

In order to assess if the profile of the “test patient” could be reproduced in any remaining patients, an unsupervised clustered analysis was performed. Figure 1 depicts expression levels of assessed genes in the whole series.

As shown, the “test patient” (lane #6), which was classified as RAEB1, with IPSS intermediate-1, and normal karyotype, tightly clustered together with patient #4 (lane #5), affected by RAEB2, and with patient #7 (lane #4), affected by RAEB1. These patients were scored as at high and intermediate-1 IPSS respectively; both showed normal karyotype and high WT1 levels.

When the gene expression profile was analyzed in the whole series, in 4 of the 6 cases with stable disease the gene expression profile did not significantly change (see patient #2, #7, #10, and #11 in Figure 1).

Three cases showed a minor response: among pro-apoptotic genes, resulted more frequently up-regulated: CASPASE-5, CASPASE-14, TNFRSF1A, HRK, BCL2L1, BCL2L14, TNFSF10, and LTB. On the contrary, BCL2L10 and BIRC8 were the anti-apoptotic genes whose expression was more frequently increased by ATO and Ascorbic Acid. In patient #1, who showed an intermediate-1 IPSS, some genes belonging to the NF-kB pathways (NFKBIA, NFKBIE, NFKBIZ, RELB) were significantly up-regulated.

Two patients achieved a major response: in the patient with RA the treatment down-regulated 69% of the pro-apoptotic genes (see case #3 in the Figure 1), whereas 91% of the pro-apoptotic genes were up-regulated in the patient affected by RAEB1 (case #8).

**Figure 2:**
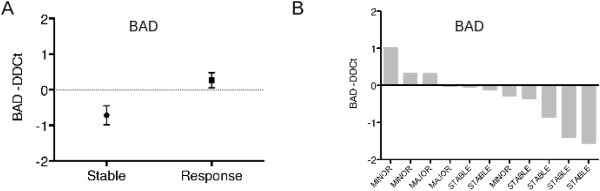
ATO and Ascorbic Acid induced gene expression modification of BAD in patients that reached stable disease or a response (major or minor).

When gene expression was further analyzed in respect of response, patients who reached a response (minor or major) showed a higher induction of BAD than those with stable disease (Figure 2).

**Figure 3:**
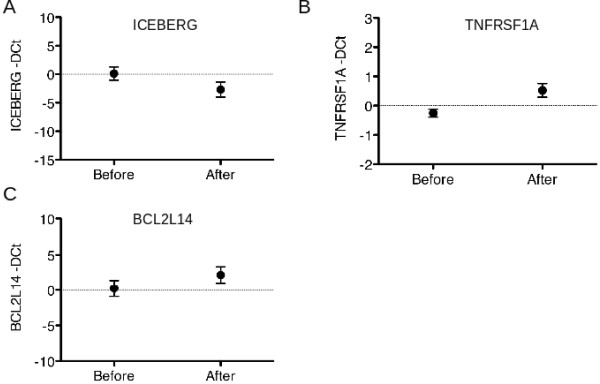
Mean and standard deviation of gene expression before and after ATO and Ascorbic Acid treatment of ICEBERG (A), TNFRSF1A (B) and BCL2L14 (C) in the whole series of patients.

Moreover, in the whole series, paired t-test demonstrated that ATO plus Ascorbic Acid induced down-regulation of ICEBERG (p = 0.0075), and up-regulation of TNFRSF1A (p = 0.0172) and BCL2L14 (p = 0.0384) in patients’ bone marrow (Figure 3 A, B and C, respectively).

Furthermore, we noticed a basal high expression of BCL2L10 in two patients affected by RAEB1 and AR, respectively. In these two patients, treatment induced a dramatic reduction of BCL2L10 expression. In the remaining cases, other 4 patients experienced a relevant reduction of BCL2L10 expression, but this reduction was not significant when the whole series was evaluated.

### Pathway analysis

**Figure 4:**
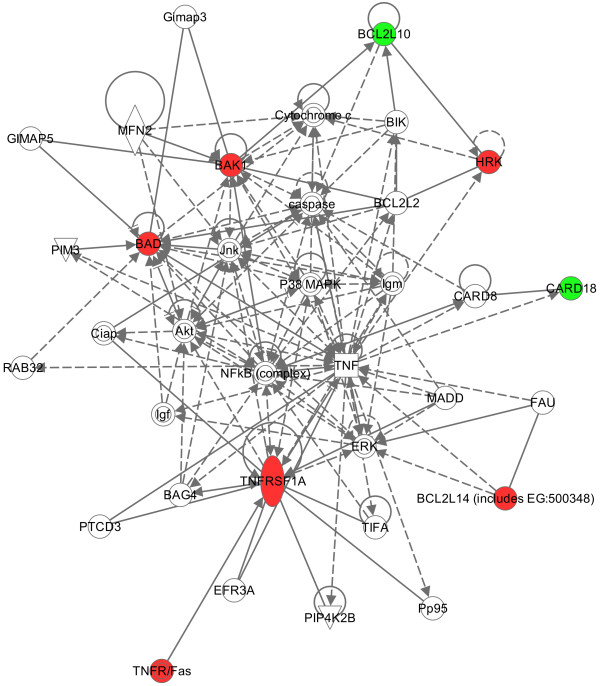
**Relationships between differentially expressed genes have been explored in the pathway analysis.** Up-regulated genes are reported in red, whereas down-regulated in green.

Ingenuity pathway analysis was adopted to explore the interaction between differently expressed genes. ICEBERG, TNFRS1A and BCL2L14 were included in the analysis because they were significantly de-regulated after treatment. BAD was included because of its higher expression in responder cases. Also BAK1, HRK and BCL2L10 were included in this analysis because of their increased values in the “test” sample (Figure 4).

### Evaluation of expression of WT1 and EVI1 genes

In the present study, we decided to assess if different values of WT1 and EVI1 measured at diagnosis and after treatment could be associated with the expression of several apoptotic genes, with particular interest in a possible common de-regulation.

At diagnosis, 10 patients showed high levels of WT1; after therapy, in 4 of them a significant reduction of the transcript was observed. Interestingly, 3 of them were the cases that achieved the major response; the correlation between reduction of the WT1 levels and good response was significant (p = 0.024).

EVI1 gene expression was detectable at diagnosis in 10 patients, 3 cases with high levels.

After therapy, 3 of them showed a significant reduction of the transcript. In the group of patients with low levels before treatment, only one showed increased levels (Table 3). No significant correlation between the behavior of the EVI1 expression and the quality of response, disease progression or death was found.

**Table 3:** WT1 and EVI1 expression levels before and after treatment and their correlation with clinical response are reported

**Pt ID**	**WT1 @ start**	**WT1 @ end**	**EVI1 @ start**	**EVI1 @ end**	**response**
**1**	high	high	low	low	**minor**
**2**	high	high	low	low	**stable**
**3**	high	normal	low	low	**major**
**4**	high	normal	low	low	**stable**
**5**	normal	normal	high	low	**minor**
**6**	normal	normal	low	low	**minor**
**7**	high	high	high	low	**stable**
**8**	high	normal	low	high	**major**
**9**	high	high	low	low	**stable**
**10**	high	high	N/A	N/A	**stable**
**11**	high	high	high	low	**stable**
**12**	high	normal	N/A	N/A	**major**

Moreover, no correlation between expression of WT1 and EVI1 (either measured at diagnosis or after therapy) and de-regulation of the pro- and anti-apoptotic assessed genes was observed.

## Discussion

It is well known that myelodysplastic syndromes are very heterogeneous entities, characterized by accelerated rates of apoptosis in early stages and higher proliferation, accumulation of less differentiated cells, with high genomic instability in the advanced phases.

Consequently, it is really difficult to derive any conclusive results from the gene expression profiling of few MDS patients. Moreover, molecular studies performed in patients receiving ATO that could increase the knowledge about real mechanisms of its action are still lacking, thus a homogenously treated series of patients, even if small, could represent a good opportunity in this field.

Here, we evaluated 12 patients affected by advanced and resistant MDS receiving ATO and Ascorbic Acid for 16 weeks. The aim was to assess ex vivo what was previously observed in vitro by our group also, with particular interest in the apoptotic pathways.

Thus, we evaluated by quantitative real-time PCR the expression of 93 genes involved in apoptosis in a “test patient” (who was analyzed just before and soon after treatment), and in other 3 patients affected by RA, 4 by RAEB1, and 4 by RAEB2.

As reported in the result section, in 4 of 6 cases with stable disease the gene expression profile did not significantly change. Two patients achieved a major response: in the patient with RA the treatment with ATO and Ascorbic Acid down-regulated about 60% of the pro-apoptotic genes, whereas, in the patient affected by RAEB, 91% of the pro-apoptotic genes were up-regulated. This observation could have a clinical impact, if we consider that in the early phases of MDS the goal of treatment would be the reduction of apoptosis and inflammatory status, whereas in the more advanced phases a perfect drug would exert principally an anti-proliferative effect.

Another interesting result from the present study is the demonstration that among principal targets for ATO there are genes belonging to the BCL2 family, analogously to that already observed in vitro. Interestingly, responsive patients showed a higher induction of BAD than those with stable disease. High expression of the pro-apoptotic BCL2-family proteins (BAK, BAD, BCL-XS, as well the apoptosis facilitator BCL2L14) has been previously associated with longer survival and decreased risk of leukemic transformation [17]. In our series, BCL2L14 expression was significantly increased by ATO and Ascorbic Acid, both in the “test patient” and in responsive cases. On the other hand, the expression of BCL2L10, an anti-apoptotic gene, resulted significantly down-regulated in 4 cases, as observed in the “test patient”.

Moreover, the expression of ICEBERG was significantly reduced in 8 cases; this gene physiologically inhibits caspase-1 activity. Caspase-1 is able to activate the IL-1β precursor; this could be interesting, because various myelosuppressive and pro-inflammatory cytokines have been implicated in the high rates of apoptosis and hematopoietic suppression seen in MDS [17,18]. Thus, decreased expression of ICEBERG could be a relevant target for new therapeutic strategies in early MDS.

Another gene that was significantly up-regulated after treatment is the TNFRSFA1 (Tumor necrosis factor receptor super family member 1A); its protein is one of the major receptors for the TNFα, and it is physiologically able to mediate apoptosis [19]. Consequently, its up-regulation could be another interesting target for treatments in advanced MDS.

Finally, a significant association between low WT1 levels and good response was found, in line with the negative prognostic role of WT1 in MDS progression previously shown [20].

Recently, immunological approaches to inactivate WT1 in acute leukemia and high-risk MDS have been proposed: these studies provided preliminary evidences of potential clinical efficacy in these patients [21].

## Conclusions

In conclusion, the present study to our knowledge is the first one that analyzed gene expression profile in a homogeneously treated series of MDS patients receiving ATO plus Ascorbic Acid. It confirmed ex vivo some mechanisms of action already demonstrated in vitro. In particular, genes belonging to the BCL2 family appeared particularly relevant in the conditioning outcome of MDS patients; this observation could translate in a possible promising therapeutic approach with BCL2 inhibitors [22].

### Ethics approval

This trial was registered as EudracT Number 2005-001321-28.

This study was conducted in accordance with the Declaration of Helsinki, and all patients gave informed consent for molecular tests.

## Abbreviations

(ATO): Arsenic Trioxide; (MDS): Myelodysplastic syndromes; (IPSS): International Prognostic Scoring System; (WPSS): WHO classification-based prognostic scoring system; (IWG): International Working Group; RA: refractory anemia; RARS: Refractory anemia with ringed sideroblasts; RCMD: Refractory cytopenia with multilineage dysplasia; RCMD-RS: Refractory cytopenia with multilineage dysplasia and ringed sideroblasts; RAEB-1: Refractory anemia with excess of blasts-1; RAEB-2: Refractory anemia with excess of blasts-2.

## Competing interests

The authors have not competing interests to declare.

## Authors’ contributions

GS designed the study, analyzed data, and wrote the manuscript; Guerrini F, Cilloni D, and Palumbo GA performed PCR assays; SF, GD, ME, LA, were principal investigators in the clinical trial; PI and PM analyzed data, and revised the manuscript. All authors read and approved the final manuscript
